# Automated confidence estimation in deep learning auto‐segmentation for brain organs at risk on MRI for radiotherapy

**DOI:** 10.1002/acm2.14513

**Published:** 2024-09-16

**Authors:** Nouf M. Alzahrani, Ann M. Henry, Bashar M. Al‐Qaisieh, Louise J. Murray, Michael G. Nix

**Affiliations:** ^1^ Department of Diagnostic Radiology King Abdulaziz University Jeddah Saudi Arabia; ^2^ School of Medicine University of Leeds Leeds UK; ^3^ Department of Clinical Oncology St James's University Hospital Leeds UK; ^4^ Department of Medical Physics and Engineering St James's University Hospital Leeds UK

**Keywords:** AI, autosegmentation, brain, confidence, deep learning, MRI scans, organs at risk, radiotherapy, uncertainty

## Abstract

**Purpose:**

We have built a novel AI‐driven QA method called AutoConfidence (ACo), to estimate segmentation confidence on a per‐voxel basis without gold standard segmentations, enabling robust, efficient review of automated segmentation (AS). We have demonstrated this method in brain OAR AS on MRI, using internal and external (third‐party) AS models.

**Methods:**

Thirty‐two retrospectives, MRI planned, glioma cases were randomly selected from a local clinical cohort for ACo training. A generator was trained adversarialy to produce internal autosegmentations (IAS) with a discriminator to estimate voxel‐wise IAS uncertainty, given the input MRI. Confidence maps for each proposed segmentation were produced for operator use in AS editing and were compared with “difference to gold‐standard” error maps. Nine cases were used for testing ACo performance on IAS and validation with two external deep learning segmentation model predictions [external model with low‐quality AS (EM‐LQ) and external model with high‐quality AS (EM‐HQ)]. Matthew's correlation coefficient (MCC), false‐positive rate (FPR), false‐negative rate (FNR), and visual assessment were used for evaluation. Edge removal and geometric distance corrections were applied to achieve more useful and clinically relevant confidence maps and performance metrics.

**Results:**

ACo showed generally excellent performance on both internal and external segmentations, across all OARs (except lenses). MCC was higher on IAS and low‐quality external segmentations (EM‐LQ) than high‐quality ones (EM‐HQ). On IAS and EM‐LQ, average MCC (excluding lenses) varied from 0.6 to 0.9, while average FPR and FNR were ≤0.13 and ≤0.21, respectively. For EM‐HQ, average MCC varied from 0.4 to 0.8, while average FPR and FNR were ≤0.37 and ≤0.22, respectively.

**Conclusion:**

ACo was a reliable predictor of uncertainty and errors on AS generated both internally and externally, demonstrating its potential as an independent, reference‐free QA tool, which could help operators deliver robust, efficient autosegmentation in the radiotherapy clinic.

## 1 INTRODUCTION

Deep learning (DL) not only promises efficiency and quality improvements for radiotherapy (RT)^1^ but also raises concerns around potential adverse safety consequences.^1^, ^2^ The most developed and commonly deployed DL application in RT is autosegmentation (AS).^3^, ^4^ Despite efficiency gains, errors and uncertainty resulting from data and model limitations necessitate time‐consuming human review and editing, which can reduce or eliminate efficiency gains.^5^ Editing is also prone to inter‐operator variability and bias, reintroducing inconsistencies, and potential segmentation errors.^5^, ^6^ Uncertainty quantification is vital to allow operators to evaluate appropriate localised confidence in AS.^2^, ^7^ It has been attempted previously, using internal probability estimates from the segmentation model itself.^5^

However, research into uncertainty estimation for AS remains nascent.^1^ Without any ground‐truth, predicting and evaluating uncertainty remains challenging.^7^

Clinical quality assurance (QA) for AS is still developing, and remains a manual process in general; however, recommendations have been made that any QA tool should be independent of the underlying AS model, rather than relying solely on internal model probabilities.^5^, ^8^

While most DL models can provide internal probabilities alongside class predictions, these are typically poorly calibrated,^9^, ^10^ with high‐probability predictions for most voxels, except those with predictions extremely close to decision boundaries. This behavior raises the spectre of “confident‐but‐wrong” predictions, which are a major concern in safety critical applications like RT.

A more useful concept than probability is uncertainty. Uncertainty can be broken down into epistemic (driven by model limitations) and aleatoric (driven by data variability) uncertainty.^1^, ^7^, ^11^ Several approaches to estimating these uncertainties have been developed, including Monte‐Carlo Dropout (MCD), ensemble‐model (EM), and Spectral‐normalized Neural Gaussian Process (SNGP).^7^, ^12^ MCD and EM predict a distribution of possible results, while SNGP integrates variance into a classifier to estimate prediction uncertainty, based on distance‐awareness.^7^, ^12^

Spatial probability maps based on MCD have been previously explored as a QA method for AS in RT, although with limited correlation between predicted uncertainty and observed error.^13^ Similarly, a secondary neural network (NN) was used to predict Dice similarity coefficient from CT‐AS pairs,^14^ with internal class probability as an input to the QA network. However, these techniques all rely on the prediction‐generating model and training data distribution to produce uncertainty estimates, and hence fail the test of independence. They are also susceptible to internal probability calibration issues as well as creating practical challenges for use with existing AS models, which may not provide probabilistic predictions.

Herein, we propose a model‐agnostic, independent uncertainty estimator, based on correlation of features in the underlying image and proposed segmentation, rather than internal AS model probability. In principle, a secondary NN can estimate errors in AS predictions, if these errors are known, relative to data labels. However, clinical confidence depends on identifying both known errors, and regions of low segmentation confidence, even if no actual error is present. We aim to combine these objectives, producing a “confidence map” that highlights both (i) regions of likely error and (ii) regions of low confidence due to either aleatoric uncertainty (data uncertainty—e.g., low image contrast and dissimilar image acquisition) or epistemic uncertainty (model uncertainty), whether they correlate with actual errors relative to labels or not. To achieve the latter, we require a distribution of AS predictions on each image, to train a secondary confidence model.

By leveraging adversarial learning, in which one NN learns to critique the predictions of another, we can address both objectives in a unified framework. Our discriminative network is trained to estimate the voxel‐wise probability that a test segmentation is derived from the gold standard population. This probability is consistently low if the AS network prediction is consistently incorrect, and highly variable if the AS network predictions varies. The network therefore learns not only to predict where discrepancies with gold standard labels occur at the sample level but also regions of low confidence, in which such discrepancies are more likely at the population level.

We present AutoConfidence (ACo), a novel AI‐driven QA method to estimate AS confidence on a per‐patient basis, without a gold standard reference. We demonstrate it for AS of brain OARs on MRI, against internal segmentations from the generator network (IAS) and external (third‐party) AS models of varying quality. ACo produces a voxel‐wise confidence map enabling efficient and robust manual verification and editing. ACo was designed to focus users’ attention on regions of error and low confidence, which require attention to avoid significant segmentation errors, potentially improving safety, confidence, and efficiency in clinical practice.

## 2 MATERIALS AND METHODS

### 2.1 Network concept and architecture

ACo was an adversarial architecture based on the generative adversarial network (GAN) concept, using two NNs, a generator and discriminator. In contrast to conventional conditional GANs (cGAN),^15^ where the discriminator exists only to help train the generator; here, the generator existed solely to provide a training example distribution of possible segmentations for the discriminator, which was used independently at imputation to provide confidence estimates on segmentations from any source.

**FIGURE 1:**
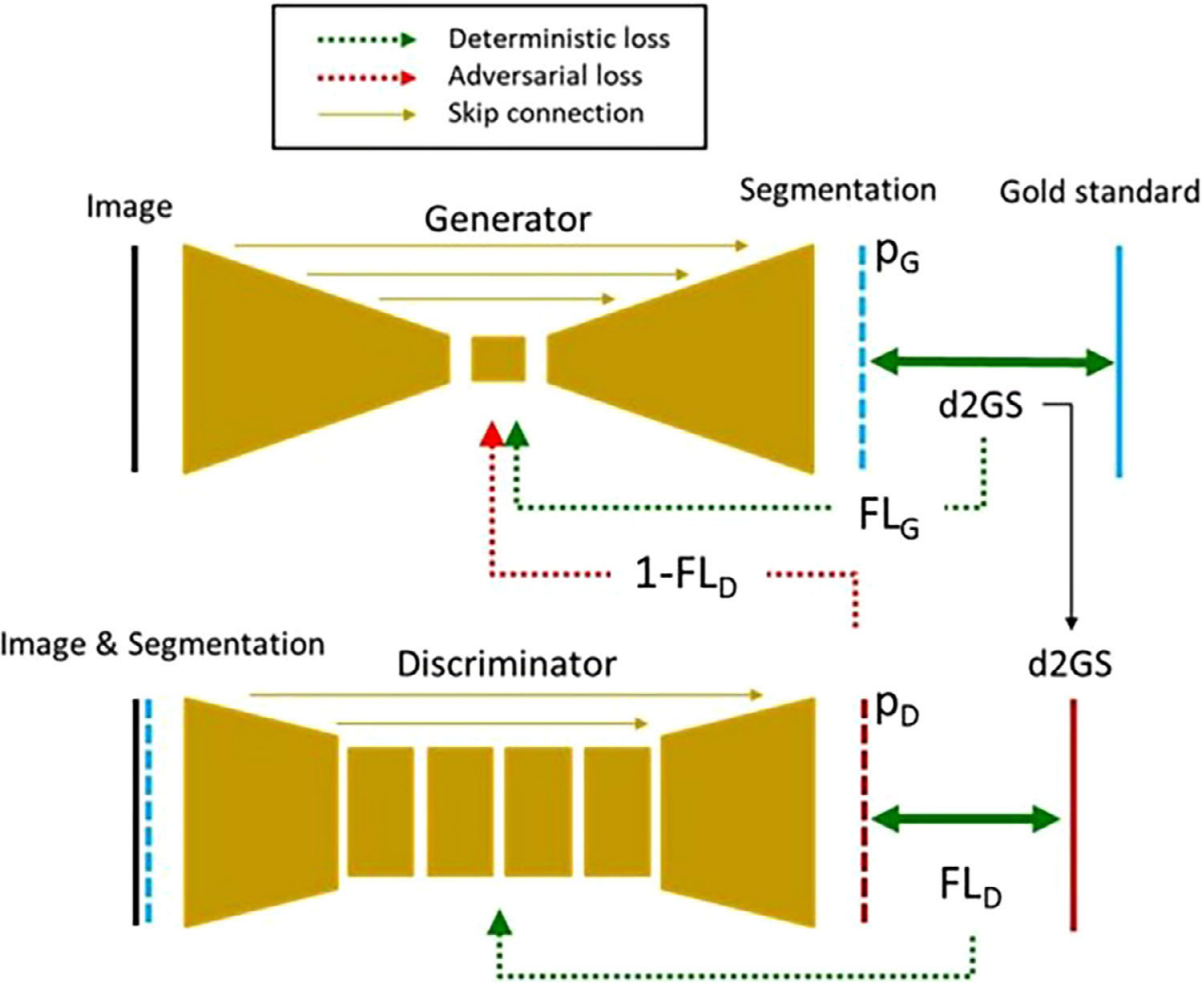
Model architecture overview for ACo, showing the segmentation generator and confidence estimator (discriminator). Segmentation (p_G_) difference to gold standard (d2GS) is used to compute the focal loss to the generator (FL_G_) and as a label for the discriminator. Confidence predictions (p_D_) are compared to d2GS labels, resulting in FL_D_, the loss to the discriminator, and 1‐FL_D_, the adversarial loss to the generator.

Medical images were fed to the segmentation generator, which aimed to produce convincing segmentations. These were fed, along with the images, to the discriminator, which aimed to estimate the voxel‐wise probability that the segmentation was correct, given the difference to the (noisy) gold standard (d2GS) as labels. The discriminator therefore estimated the probability that a segmentation was “plausible” for a given image slice (Figure 1).

The generator was a typical 2D‐U‐net segmentation network with eight convolutional layers, featuring batchnorm (BN), dropout, and “relu” activation. The generator took 2D MRI slices as input and predicts segmentations per voxel, with softmax activation at the final layer. Focal log loss (γ = 2) against gold standard segmentation labels was used as the non‐adversarial loss:

$$FL_{G}\;\left(p_{g}\right)=-y.{\left(1-p_{g}\right)}^{\gamma }\log\left(p_{g}+\varepsilon \right)$$

Here, $p_{g}$ was the probability in the target class, y was the label probability in the target class (which was always unity) and γ was the focussing parameter. ε represented a small offset to prevent infinite loss for $p_{g}=\;0$.

In further contrast to cGANs, the discriminator followed a U‐RESnet architecture, extending the concept of a patch‐wise discriminator to a shallow encoder‐decoder, providing voxel‐wise discrimination, with residual blocks to improve performance in the shallow architecture. The discriminator took multichannel input, with each OAR represented as a single channel binary mask and the MR slice as a final channel and learned to predict voxel‐wise difference to gold‐standard (d2GS) as a surrogate for pGS. d2GS was a single‐channel binary mask, computed as the channel‐wise maximum of the absolute difference between predicted binary segmentation masks and gold‐standard binary segmentation masks. The output of the discriminator was a softmax probability of each voxel belonging to the error or correct class (in d2GS). A voxel that was under‐segmented or over‐segmented by the AS model was labeled as 1 in d2GS binary mask and belongs to error class.

The discriminator discriminated predicted segmentations from gold standard segmentations (GS), on a per‐voxel basis, with focal log loss (as above) against d2GS labels, which is provided as a binary voxel‐wise label mask.

$$FL_{D}\;\left(p_{d}\right)=-y.{\left(1-p_{d}\right)}^{\gamma }\log\left(p_{d}+\varepsilon \right)$$

Here, $p_{d}$ was the predicted probability in the target (error or non‐error) class, y was the label probability in the target class (which was always unity), and γ was the focussing parameter. ε represented a small offset to prevent infinite loss for $p_{d}=0$.

The discriminator was trained alternately with gold standard contours and AS predictions, for each input image. The generator and the discriminator were updated alternately for each batch during training. The total generator loss was therefore:

$$L_{gen}=\alpha .FL_{G}\left(p_{g}\right)+\beta .\left(1-FL_{D}\left(p_{d}\right)\right)$$

where α and β were loss weighting factors equal to 20 and 1, respectively. The total discriminator loss was simply $FL_{D}\left(p_{d}\right).$

The output of the discriminator therefore represented a confidence map, across the segmentation, which was used as an adversarial loss to augment training of the generator. This generator adversarial loss $1-FL_{D}$ (discriminator focal log loss), on the generated sample, resulting in a typical min‐max GAN optimisation scheme. This loss encouraged the generator to modify AS predictions to increase confidence, where the discriminator predicted low confidence. Hence, errors were reduced, making the task more difficult and consequently improving the performance of the discriminator on hard examples. Detailed model architecture and hyperparameters are provided in Supplementary information (research method).

### 2.2 Data and training

ACo models were trained using 32 retrospective, clinical glioma cases, each containing ∼100 clinical T1‐weighted gadolinium‐enhanced MRI (T1w‐Gd MRI) slices, voxel size 1*1*2 mm, with gold‐standard contours, manually quality assured by editing clinical contours. Nine further cases were used for validation. Ethical approval for retrospective use of de‐identified patient data was given by Leeds East REC, reference: 19/ YH/0300, IRAS project ID: 255 585.

Briefly, MRI was T1w spin echo, imaging plane: transverse, slice thickness: 2 mm, scanner: Siemens Magnetom Sola with 1 mm in‐plane resolution and Gd contrast. OAR selection was based on the four central nervous system clinical protocols at our institution: Meningioma, Pituitary, Glioma (Radical), and Glioma (Palliative). Thirteen OARs were selected for AS and confidence prediction: brainstem, cochlea (left and right), orbits (left and right), lenses (left and right), optic chiasm, optic nerves (left and right), lacrimal glands (left and right), and pituitary gland. Contouring was performed in line with international consensus guidelines. Details concerning image acquisition, OARs selection, and gold standard contour delineation are found in our previous published work.^16^

ACo was initially trained as described above. A second, otherwise identical, model was trained by adding synthetic errors to the IAS produced by the generator, before the discriminator estimated pGS. These included random geometric deformations (rotation, translation, and non‐rigid deformation based on thin‐plate splines with a normal distribution of deformation vectors generated on a 2 cm regular grid), random OAR class perturbations (altering the label of a predicted OAR to that of the next most likely under softmax), and random removal of an entire OAR mask. These synthetic errors were intended to mimic potential real‐world errors from AS algorithms.

### 2.3 Evaluation of AutoConfidence performance

Nine independent glioma test cases were used to evaluate the performance of both models on the IAS. The following metrics were used to compare the outputs of the ACo map that was produced by each model relative to the d2GS: using true positive count (TP), true negative count (TN), false‐positive count (FP), and false‐negative count (FN), Matthew's correlation coefficient (MCC), false‐positive rate (FPR), false‐negative rate (FNR), FP/FN ratio. Note that these metrics compare the predicted error maps to the d2GS gold‐standard error maps rather than the more typical direct comparison of predicted to ground‐truth segmentations.

Two corrections [intelligent edge removal (IER) and geometric distance correction(GDC)] were applied to the model output, and their impact was assessed using the same methodology. The corrections were justified, applied, and optimized as follows.

#### 2.3.1 Intelligent edge removal (IER)

**FIGURE 2:**
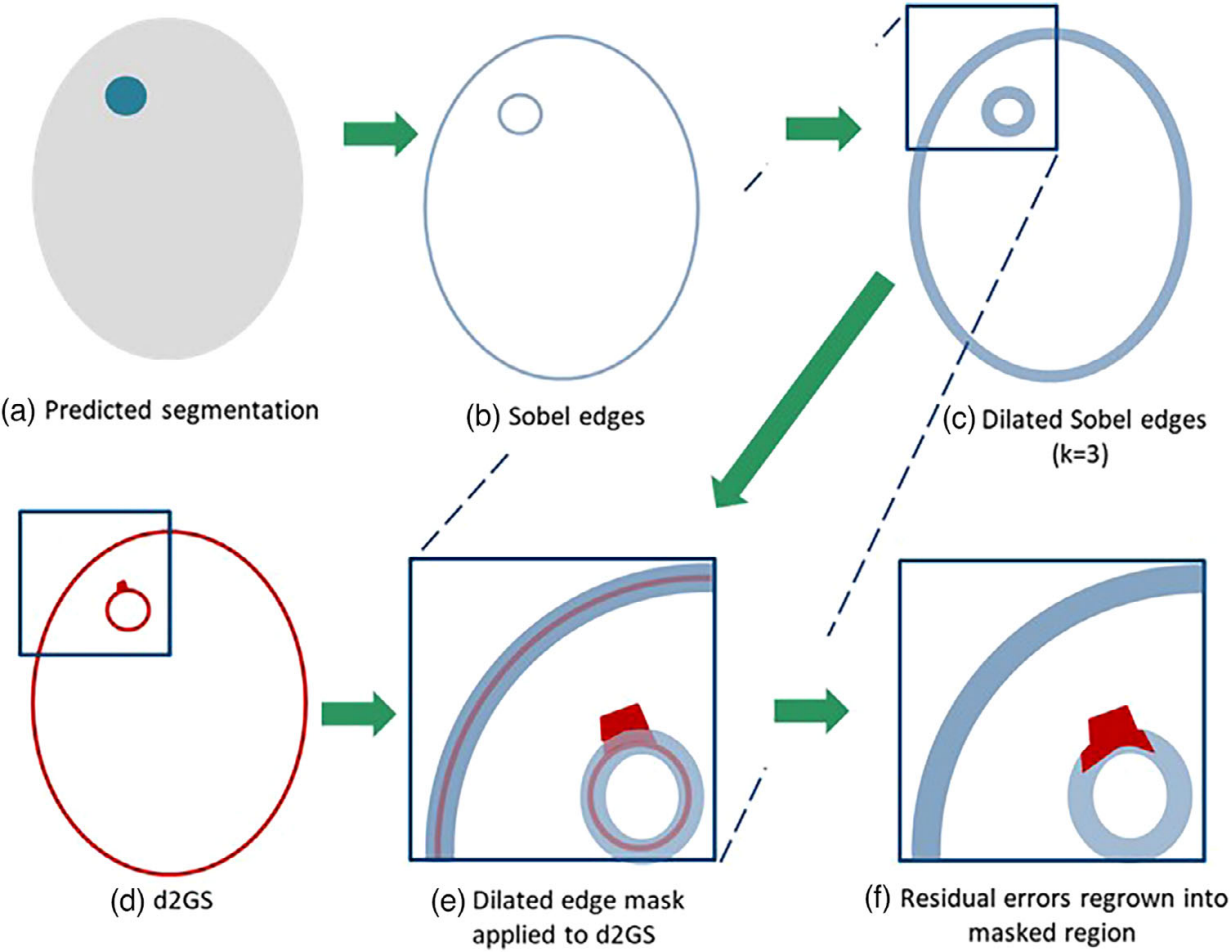
IER algorithm. Sobel edges (b) were generated from predicted segmentations (a) and dilated (c) with a kernel size of 3 voxels. d2GS (d) was masked with the dilated edges (e) to remove errors at the OAR boundaries. Finally, remaining errors were regrown into the boundary region (f).

IER was applied to remove “thin” (clinically insignificant) error regions around OAR borders, which resulted from partial volume effects at the edges of binary segmentation masks. Sobel edges of the segmentation were computed using the SciPy 1.12 package and dilated to a width of k voxels. This edge mask was applied to the confidence map and d2GS map, removing errors close to OARs boundaries. Errors remaining after this process were regrown by k/2 voxels back into this region, to avoid removing significant errors adjacent to OAR boundaries (Figure 2). Visual assessment was used to determine the optimal mask width as k = 3, as the minimum value which resulted in removal of thin error predictions along all OAR boundaries.

#### 2.3.2 Geometric distance correction (GDC)

ACo was designed to detect gross segmentation errors and also flag areas of low confidence in the underlying AS. The data used for ACo training, d2GS, contains only the gross segmentation error. If we employ just d2GS as reference to evaluate ACo performance, the areas of low confidence would appear as FP, even though they were clinically meaningful. Therefore, we proposed GDC, a post‐processing method, to account for the effect of high uncertainty when we evaluate ACo against d2GS. Please note that this GDC correction was applied only at the level of the evaluation metrics, producing more meaningful estimates of ACo performance. The predicted confidence maps themselves, which are the output presented to clinicians to aid contour review were completely unaffected by this correction.

Low confidence regions (e.g., where image contrast is low) are typically geometrically close to actual error regions, but extend beyond them. Hence, apparent FP prediction errors are often actually regions of low confidence, which are correctly highlighted by ACo but absent from the ground‐truth labels.

We used proximity to TP as a metric for when an apparent FP voxel should be considered correct, and when it likely represented a genuinely false positive prediction (i.e., far from any real error or low confidence region). When a FP voxel was “close” to a sufficiently large true positive region, it was considered clinically useful as an indicator of potential error (i.e., low confidence), and counted as TP for Aco performance evaluation. Our definition of “closeness” depended on the size of the true positive region within a local image patch, which should be larger than the distance to it from the voxel in question.

Similarly, if a small FN voxel region was close to a large TP region, it could be considered unimportant, as the TP region would draw operator attention clinically. However, if a FN region appeared without a large proximal TP region, it would be significant as a missed region of error or low confidence, so should not be reclassified to TN.

To make the quantitative performance analysis more clinically relevant, prediction classes were therefore updated as follows: Within a local patch of the image, the maximum dimension of the TP region [D(tp)_max] was computed via Euclidean Distance Transform (EDT). Second, the minimum distance to the TP region from the central voxel [D(x_tp)] was computed, also via EDT. If D(x_tp) <D(tp_max) and the central voxel was either FP or FN, it was reclassified as TP or TN respectively (see Figure S1).

Importantly, this geometric correction was applied only at the level of the evaluation metrics, producing more realistic estimates of ACo performance in the absence of a complete ground truth. The predicted confidence maps themselves, which are the output presented to clinicians to aid contour review were completely unaffected by this correction.

### 2.4 Validation with external autosegmentation

The same nine test cases were used to evaluate the performance of ACo model on external MRI AS that generated using custom models based on a commercial 3D medical image segmentation U‐net (RayStation 11A, RaySearch AB). Two external models were used; external model—low quality (EM‐LQ) and external model—high quality (EM‐HQ), which were previously trained, using clinical and gold standard contours respectively, allowing evaluation of AutoConfidence in both scenarios. These models were trained on the same MR image dataset used herein, and the EM‐HQ model was trained using carefully edited gold‐standard segmentations to provide clinically acceptable results. The EM‐LQ model was trained on unedited clinical contours, which contained more variability and errors than the curated dataset, and was used here as an example of a lower performing model which might generate clinically significant segmentation errors (*More information about the difference between these models can be found in our previous work*.)^16^

Evaluation was done pre and post‐application of IER and GDC, as described above.

“Four‐colour maps” were produced for visualisation of FP, TP, FN regions, and the regions modified by GDC. Visual assessment was performed by three expert observers, to evaluate the clinical utility of the ACo model predictions, based on the location, type, and appropriateness of regions highlighted as low confidence, especially where no explicit errors existed in the d2GS reference.

## 3 RESULTS

### 3.1 Internal autosegmentation quality

Relative to gold standard, the generator produced acceptable IAS, with average test DSC from 0.47 to 0.85 for all structures except lacrimal glands (DSC ≤0.30) (Table S1).

#### 3.1.1 Internal versus external autosegmentation quality

Excluding lacrimal glands, IAS performance (DSC 0.47–0.85) was comparable to EM‐HQ (DSC 0.49–0.91) (Table S1). IAS outperformed EM‐LQ (DSC 0.28–0.89). All models performed equally poorly for lacrimal glands (DSC ≤0.26).

### 3.2 AutoConfidence results

#### 3.2.1 AutoConfidence outputs on the internal AS

ACo trained with synthetic errors was superior to ACo without synthetic errors. FNR, using d2GS as a reference, showed a mean reduction of 7% across 11/13 OARs (Tables S2 and S3), using synthetic errors. FPR was not strongly affected (mean ΔFPR = 0.4%). Mean MCC improved by 0.075. All subsequent ACo results were obtained with synthetic errors during model training.

**FIGURE 3:**
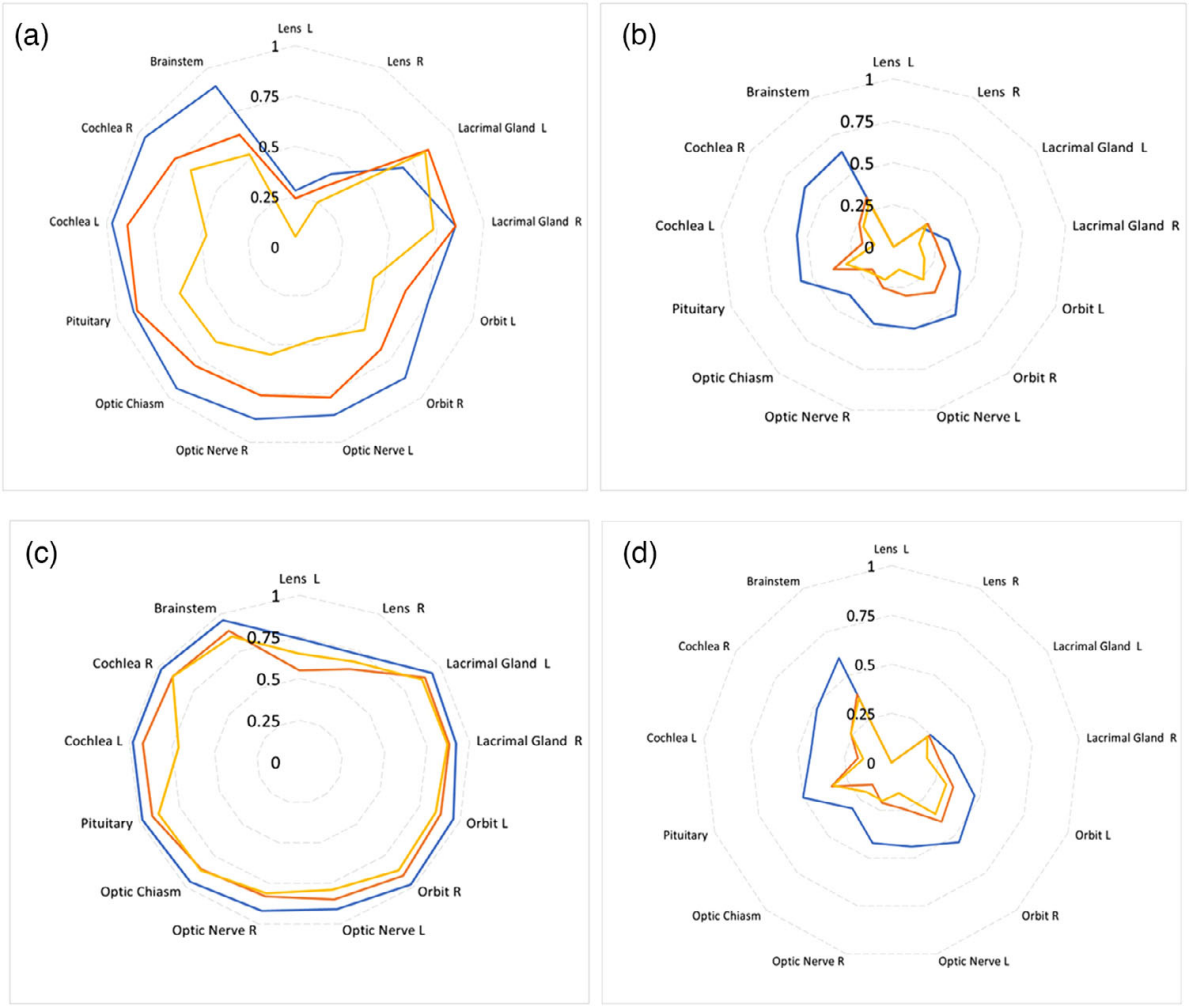
Mean MCC for AutoConfidence per OAR and per segmentation model. Performance is shown on IAS (blue), MRIu (orange), and MRIeMRI (yellow) segmentations. (a) MCC of ACo with IER and GDC combined, (b) IER only, (c) GDC only, and (d) baseline ACo output without corrections.

MCC on IAS, following application of IER and GDC, was > 0.69 (Figure 3a) for all OARs except lenses (MCC ≤0.40). FPR and FNR on IAS were ≤0.13 and ≤0.17, respectively (≤0.53 and ≤0.16 for lenses, resulting in a high FP/FN ratio of 9.0 (Table S4).

ACo performance was shown to rely on both MRI and AS inputs via ablation testing. Removing either images or segmentations during training and testing severely affected confidence estimation (mean MCC 0.05 and 0.08, respectively). Furthermore, training IAS generator and ACo networks sequentially, as opposed to adversarially, severely impacted ACo test performance (mean MCC <0.23).

#### 3.2.2 AutoConfidence outputs on the external AS

**FIGURE 4:**
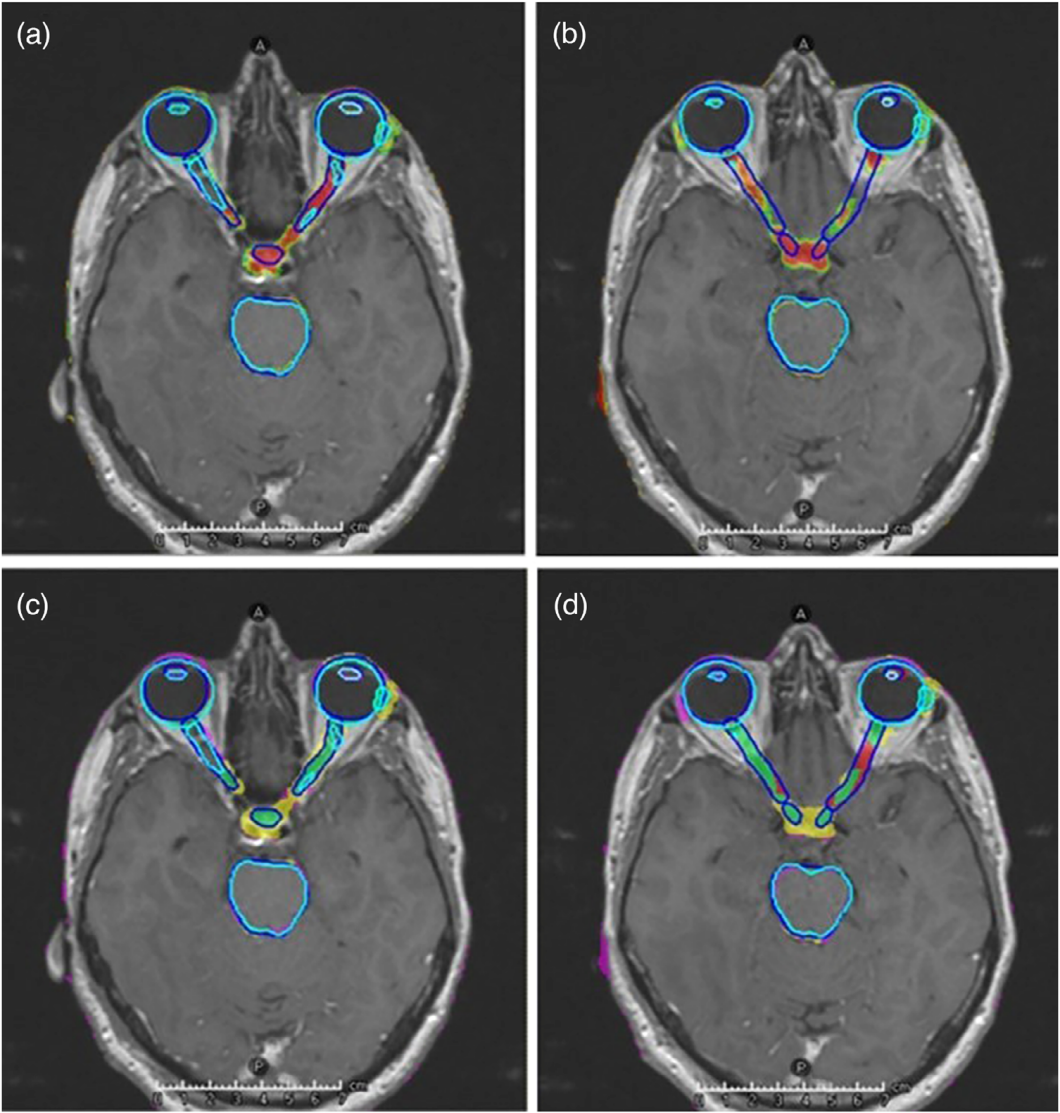
Axial T1w‐Gd MRI with dark‐blue contours representing gold standard and light‐blue representing EM‐LQ autosegmentation. (a) Example showing a high uncertainty level for missing pituitary segmentation and errors in optic nerves. (b) High uncertainty for missing optic chiasm and apparent false‐negative predictions for optic‐nerves, which are in fact due to non‐anatomical GS contours (see main text). FP in the region near lacrimal glands is typical of ACo on MRI, where lacrimal glands are very hard to visualize and therefore highly uncertain in location. (c) and (d) Four‐color‐map showing regions of TP (green), FP (pink), and GDC modified FP (yellow) relative to the differences‐to‐gold‐standard, for the ACo prediction.

An example of the ACo output confidence map and a color‐coded comparison to d2GS is shown in Figure 4. Following IER and GDC, and excluding lenses, mean MCC for ACo on EM‐LQ ASs ranged from 0.62 to 0.89, with left orbit, right orbit, and brainstem having the lowest scores (0.62, 0.68, and 0.63, respectively), and a high error ratio (FP/FN ≥2.13) (Figure 3a, Table S5). Mean MCC for ACo on the EM‐HQ ASs ranged from 0.44 to 0.83, with left orbit, left cochlea, and left optic nerve having the lowest scores (0.44, 0.47, and 0.47, respectively), and FP/FN ≥2.67 (Figure 3a, Table S6).

The lowest mean MCC scores for ACo across all the Raystation MRI AS models were for lenses (average MCC ≤0.34) (Figure 3a). FP/FN for lenses on EM‐LQ was ≤0.67, but for EM‐HQ was 3.0 (Tables S5 and S6).

### 3.3 Impact of postprocessing on ACo

#### 3.3.1 Intelligent edge removal (IER)

IER alone had limited impact on quantitative ACo performance. Mean MCC improved by 0.03 and 0.01 compared to baseline for IAS and EM‐LQ AS, respectively, and reduced by 0.04 for EM‐HQ AS (Figure 3b).

Mean FPR showed a reduction of 0.09, 0.08, and 0.11 relative to baseline, on IAS, EM‐LQ, and EM‐HQ AS, respectively. Absolute FP count was also reduced for all OARs, driving the change in FPR.

Mean FNR increased by 0.06, 0.06, and 0.14 on IAS, EM‐LQ, and EM‐HQ ASs, respectively (Tables S7–S9). Absolute FN count did not increase significantly for any OARs. TP count decreased significantly for Orbits and Brainstem on internal and EM‐LQ ASs. For EM‐HQ, all OARs exhibited significant decreases in TP count. These changes in TP count drove the observed changes in FNR (Table S9).

Mean FPR, FNR, and MCC for the ACo outputs using the IER can be found in Tables S7 and S8.

#### 3.3.2 Geometric distance correction (GDC)

GDC improved mean MCC across all OARs by 0.50, 0.63, and 0.62 for IAS, EM‐LQ, and EM‐HQ AS, respectively, relative to the baseline (Figure 3c).

GDC reduced both FPR and FNR relative to baseline across all OARs and AS models. Performance on IAS, EM‐LQ, and EM‐HQ showed reductions of 0.35, 0.38, and 0.32, respectively. This change in FPR was directly driven by corresponding changes in FP count (see Tables S10–S12).

Mean FNR showed a reduction with GDC of 0.19, 0.22, and 0.24 on the IAS, EM‐LQ, and EM‐HQ AS, respectively. These changes were driven in part by small reductions in FN count but were predominantly a result of increased TP count.

Mean FPR, FNR, and MCC for ACo using GDC can be found in Tables S10–S12.

#### 3.3.3 Combined corrections (IER and GDC)

Combining the two postprocessing corrections improved mean MCC across all OARs by 0.43, 0.49, and 0.33 relative to baseline for IAS, EM‐LQ, and EM‐HQ AS, respectively (Figure 3a).

IER and GDC combined decreased mean FPR relative to baseline for all OARs by 0.27, 0.33, 0.23 for IAS, EM‐LQ, and EM‐HQ AS, respectively. Mean FNR on IAS, EM‐LQ, and EM‐HQ AS was reduced by 0.15, 0.14, and 0.11, respectively.

The mean FPR and FNR for baseline and for combined corrections can be found in Tables S4–S6.

## 4 DISCUSSION

There is strong demand in RT for deep learning OAR and target AS to improve efficiency. However, efficiency gains can be eroded by the need for time‐consuming manual checking and editing. Robust automation is desirable but challenging due to lack of automated segmentation QA tools. Our novel AI‐driven QA tool (AutoConfidence) can estimate segmentation confidence from the underlying image and proposed segmentation, on a localized, independent, per‐patient basis, without gold standard reference segmentation. Our approach is based on adversarial generative learning, utilizing the errors and variability of an internal segmentation NN to train an independent discriminative network to predict a map of segmentation confidence, which is then used to optimize the segmentation model and adversarially train both models. ACo can estimate confidence for segmentations from any source (including manual contours).

Importantly, ACo was designed to highlight regions of low AS confidence to the user. These may correspond to delineation errors, relative to gold standard or represent regions of correct segmentation relative to gold standard, but low confidence due to low contrast, high variability in gold standard contouring, or imaging artefact. ACo does not distinguish between areas of (e.g.) low contrast and likely errors (to ground truth), rather the model highlights all regions of low confidence equally for human review. In either situation, ACo will attract operator attention to QA the contour in the suspect region.

Due to lack of ground‐truth for segmentation confidence as defined above, quantitative analysis of ACo performance was challenging. As a surrogate, we used “difference‐to‐gold‐standard” (d2GS), the difference between autosegmented contours and gold standard human contours. However, this surrogate is imperfect for two reasons. First, gold‐standard contours can be erroneous, such that apparent false‐negative or false‐positive regions can derive from label inaccuracy rather than prediction error. Second, d2GS only accounts for regions of actual difference between prediction and gold standard, whereas ACo also identifies low‐confidence regions, even when the prediction and gold standard align perfectly.

ACo showed generally excellent performance on both internal and external segmentations, across all OARs except lenses (Figure 3, Tables S4, S5, and S6), once IER and GDC were applied.

IER removed thin regions of low‐confidence predictions within 2 pixels of an OAR boundary, improving visual perception and enabling the operator to focus on more significant low‐confidence regions.

IER (w = 3) reduced average FPR by ∼0.1 across all MRI AS models and OARs (Tables S7–S9), making the ACo map more useful and clinically relevant.The IER algorithm “re‐grows” larger low‐confidence signals into the boundary region, ensuring clinically significant predictions were not adversely affected.

The observed increase in FNR, between 0.06 and 0.11, was primarily driven by removal of TP voxels near the OAR boundaries. This effect was especially pronounced for orbits and brainstem, which were generally well segmented, leaving the majority of residual error voxels at OAR boundaries. Hence, removal of predicted uncertainties along OAR boundaries via IER was safe and should aid visual interpretation of ACo maps.

GDC did not affect the predicted confidence maps, but rather modified the statistical performance metrics against the surrogate labels (d2GS), to better correlate with clinical significance when validating ACo. GDC accounted for the fact that not all incorrectly predicted (FP and FN) voxels were clinically equivalent. As ACo was designed to highlight low confidence (potential error) regions to human operators for review, it was considered acceptable or even desirable to predict larger regions of low confidence than represented by d2GS labels, provided they were in appropriate locations and not grossly oversized. Likewise, it was acceptable to have small regions of FN, if they were proximal to larger TP regions, as the human operator's attention would be drawn to this area anyway.

GDC strongly reduced FPR and FNR across all OARs and segmentation models. The reduction in FPR showed that most FP voxels were near significant TP regions.

For the optimized ACo model (with IER, GDC, and synthetic training errors), MCC was higher on low‐quality external segmentations (EM‐LQ) versus High‐quality ones (EM‐HQ) (Figure 3a). This was partially due to the dependence of MCC on class‐imbalance (fewer errors to detect) and also due to the higher quality model making more subtle errors, which were harder to identify. Furthermore, imperfect segmentation (and hence d2GS) labels at the voxel level represent a theoretical limit to apparent ACo performance, which will be approached as AS quality improves. The low mean FPR, FNR, and a high FP/FN ratio on EM‐HQ AS, indicated the method was safe, as it tended to be over‐cautious (FP/FN > 1), and could be used clinically (except for lenses) (Table S6). As expected, ACo performed slightly better on internally generated test segmentations, as the errors present were generated by the AS model used during adversarial ACo discriminator training.

Visual assessment by expert oncologists indicated ACo was able to successfully identify inaccurate segmentations and missing structures that generally correlated well with errors to gold standard, as well as regions of low confidence associated with poor image contrast or artefact, highlighting them for clinical review. Figure 4a–c show examples of successful error detection in the optic nerves and the missing pituitary gland. The ACo confidence map (Figure 4a) shows regions of low confidence, while Figure 4c shows the graphical confusion matric map, highlighting TP, FP, and FN voxels relative to gold standard.

**FIGURE 5:**
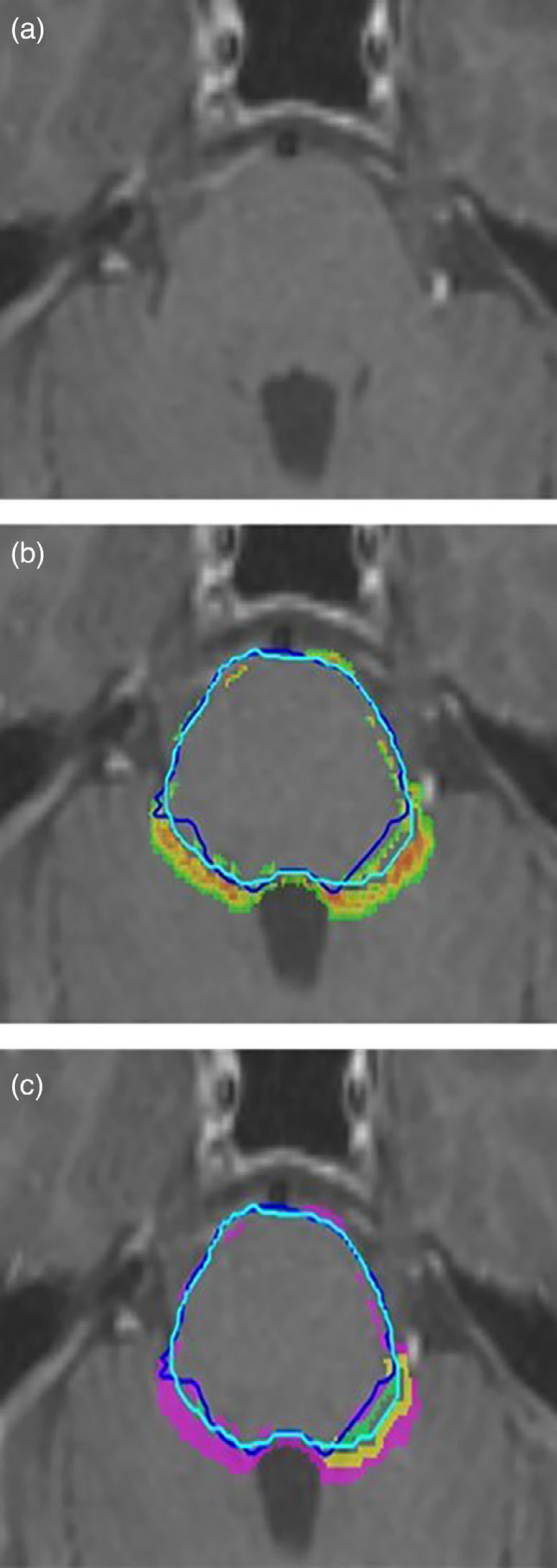
Axial T1w‐Gd MRI illustrating (a) a low contrast in the brainstem region, (b) ACo prediction (heat map), EM‐HQ segmentation (light‐blue), and gold standard (dark‐blue), showing uncertainty due to the low contrast region around brainstem. (c) Four‐color‐map showing regions of TP (green), FP (pink), and GDC modified FP (yellow) relative to the differences to gold standard, for the ACo prediction.

Where ACo predictions did not correlate with d2GS, visual inspection often revealed a systematic difference due to gold standard contouring definitions. For example, local protocol for manual optic nerve segmentation indicates that nerves should be continuous on each slice, which does not necessarily align with anatomical reality. Hence, the gold standard was not a good comparator in these regions, leading to apparent underperformance of ACo, which appropriately identified errors based on the 3D anatomical image features (Figure 4b–d). Additionally, low‐contrast regions (e.g., boundary of brainstem ‐ Figure 5) or image artefacts often led to low‐confidence predictions by ACo, in the absence of error to gold standard. This low confidence was deemed clinically appropriate, as there was genuine uncertainty in such regions.

Visual inspection also confirmed that IER made the final confidence maps more useful by removing unimportant but visually distracting low confidence predictions around OAR boundaries. While partial volume effects in the axial plane were removed via IER, both the ACo model itself and the IER algorithm operated in 2D. Hence, the superior and inferior limit of orbits and the cranio‐caudal extent of the optic nerves exhibited some residual errors from these partial volume effects, which were not removed by IER, leading to under‐prediction or over‐prediction of error in these regions. This could in principle be addressed by a 3D ACo and IER algorithm.

Interestingly, ACo detected uncertainty in erroneous regions of the external segmentation, even though it was not exposed to such errors during training. The generator did not predict external segmentations, and gold standard ones were used as input to the ACo discriminator. Nevertheless, ACo learned how external contours should look relative to images, based on many gold standard external segmentations/image pairs. Any test external‐segmentation region, which departed from this expectation was labelled as uncertain. Thus, in the unseen error scenario, ACo operated as a “zero‐shot outlier detector,” flagging previously unseen error types based on dissimilarity to the gold‐standard distribution (low pGS), potentially improving robustness in clinical use.

**FIGURE 6:**
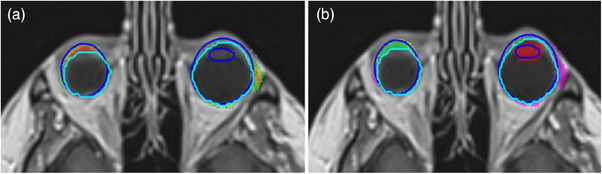
Axial T1w‐Gd MRI showing the failure of AutoConfidence prediction to detect the missing autosegmentation for the left lens, resulting in false‐negative. Blue represents the gold standard, while light blue represents EM‐HQ autosegmentation. (b) Four‐color‐map showing regions of TP (green), FP (pink), and GDC modified FP (yellow) relative to the differences‐to‐gold‐standard, for the ACo prediction.

Despite generally excellent performance of ACo, there were limitations. ACo used a 2D recurrent‐Unet, due to GPU memory constraints, whilst segmentations are inherently 3D, leading to over‐prediction or under‐prediction of errors at superior and inferior OAR limits. Furthermore, as IER was also a 2D algorithm, these issues were not mitigated by post‐processing. Lastly, the ACo model trained with only 32 cases (∼4500 slices), due to limited data availability for brain MRI. Relatively consistent cranial anatomy made this acceptable, but for a clinical site with greater anatomical variation, more data would be required. This effect was demonstrated by the poor performance of both IAS and ACo for lenses (Figure 6), which exhibited greater motion artefact and variability than any other OAR in the brain.

Also, ACo struggled to determine the inferior limit of brainstem (see Figure S2). This limit was landmark driven, defined by the tip of the dens of C2, which was not directly adjacent to brainstem, making it difficult to learn from 2D image data alone. The performance of ACo could potentially be improved in this regard by segmenting the dens, or by a 3D ACo method. A 3D version of ACo is being investigated and will potentially resolve many of these limitations.

ACo is a model‐agnostic, fully independent estimator of potential errors and combined (aleatoric and epistemic) uncertainty for medical image segmentation, at a per‐voxel, per‐prediction level. This approach is different to previously published DL AS QA methods based on internal DL‐model probability, or Monte Carlo dropout.^5^ The advantage of our approach, which does not require any additional information from, or access to, the segmentation model, is both its generalisability (deep learning, atlas‐based, or even manual contours can be assessed by ACo) and its independence. Furthermore, internal probability estimates from deep learning classifiers are notoriously poorly calibrated, typically resulting in high confidence predictions for all voxels, except very near decision boundaries, leading to the model being “confident but wrong,” a scenario one would like to avoid in safety critical medical applications.

## 5 CONCLUSION

ACo was able to successfully predict regions of low confidence, including errors to gold standard, in both internal and external AS, from a commercially available segmentation algorithm, without reference to the gold‐standard segmentations themselves.

These confidence estimates did not depend on the internal confidence of the segmentation model. Indeed, they require only the proposed segmentation and underlying image, making them fully independent and applicable to segmentations from any source, including manual delineations.

ACo confidence maps could serve as a per‐patient, reference‐free segmentation QA tool, increasing clinical confidence in AS and potentially reducing editing time, whist improving patient safety. The additional ability to detect error types of unseen during training (zero‐shot detection) enhances the robustness of ACo for clinical use.

## AUTHOR CONTRIBUTIONS

Nouf Alzahrani was responsible for collecting, preparing, and analysing the data, model training and testing, interpreting the results, and writing the manuscript. Michael Nix provided essential guidance for the study design. Louise Murray and Michael Nix provided essential guidance for reviewing the analysis and interpretation of the result. Ann Henry and Bashar Al‐Qaisieh contributed to reviewing and approving the study design from the clinical and technical perspectives and providing the overall guidance of the project and data. All authors contributed to the review of the manuscript, and all approved the final draft for submission.

## CONFLICT OF INTEREST STATEMENT

The authors declare no conflicts of interest.

## Supporting information



SUPPORTING INFORMATION
