# Supplementary material for: Automated confidence estimation in deep learning auto‐segmentation for brain organs at risk on MRI for radiotherapy
Source: J Appl Clin Med Phys. 2024 Sep 16;25(12):e14513. doi: 10.1002/acm2.14513 (PMC11633808; doi:10.1002/acm2.14513)
Supplement: Supplementary file 1 — SUPPORTING INFORMATION [file ACM2-25-e14513-s001.docx]

**Supplementary information:**

- **Research method:**

The generator is a U-net neural network that contains downsampling and upsampling paths and a Skip connection. The downsampling path includes 8 layers each has 2D Convolution layers with a rectified linear unit (*ReLU*) activation and batch normalization (batch norm). The upsampling path includes 8 layers, which 7 of have transposed 2D convolutional layer, drop out layer, and batch norm. The final layer contains transposed 2D convolution. Skip connection enhances the output of the downsampling to upsampling layers.

The discriminator is a shallow U-net neural network that contains downsampling, upsampling paths and a series of residual blocks skip connection. The downsampling path includes five layers with each having 2D Convolution layer with *ReLU*activation and batch norm. The upsampling path includes 4 layers with each having transposed 2D convolution with *ReLU*activation, drop-out layer, and batch norm.

Five blocks residual skip connection in each layer with block contains two 2D convolutional neural layers, *ReLU*activation, drop out layer, and batch norm. The output of the residual blocks it’s the output of its convolutional neural layers and the output from downsampling.

The combination of residual network architecture with U-net architecture in the discriminator is to have the ability to classify in a high voxel resolution.

The hyperparameters for training were set as follows: Batch size = 4, Filters on initial convolution layer = 16 for both generator and discriminator, weight = 100 for generator deterministic loss, weight = 1 for generator adversarial loss, gamma = 2 for focal loss, ADAM learning rate 0.0001, beta1 = 0.5, beta 2 = 0.999, dropout ratio = 0.25.

- **Tables:**

|  | Brainstem | Cochlea | Cochlea | Lacrimal | Lacrimal | Lens | Lens | Optic Chiasm | Optic | Optic | Orbit | Orbit | Pituitary |
| --- | --- | --- | --- | --- | --- | --- | --- | --- | --- | --- | --- | --- | --- |
|  |  | L | R | L | R | L | R |  | Nrv L | Nrv R | L | R |  |
| IAS | | | | | | | | | | | | | |
| Average | 0.85 | 0.52 | 0.47 | 0.07 | 0.26 | 0.77 | 0.51 | 0.60 | 0.68 | 0.63 | 0.81 | 0.78 | 0.73 |
| EM-HQ | | | | | | | | | | | | | |
| Average | 0.9 | 0.57 | 0.49 | 0.1 | 0.15 | 0.68 | 0.67 | 0.51 | 0.65 | 0.68 | 0.9 | 0.91 | 0.67 |
| EM-LQ | | | | | | | | | | | | | |
| Average | 0.89 | 0.73 | 0.52 | 0.04 | 0.02 | 0.41 | 0.28 | 0.44 | 0.41 | 0.43 | 0.88 | 0.86 | 0.35 |

*Table S1: Average DSC across OARs and segmentations.*

|  | Lens  L | Lens  R | Lacrimal Gland  L | Lacrimal Gland  R | Orbit  L | Orbit  R | Optic Nerve  L | Optic Nerve  R | Optic  Chiasm | Pituitary  Gland | Cochlea  L | Cochlea  R | Brainstem |
| --- | --- | --- | --- | --- | --- | --- | --- | --- | --- | --- | --- | --- | --- |
| TP | 4.30 | 4.28 | 10.34 | 10.19 | 55.83 | 63.24 | 14.21 | 15.79 | 9.21 | 4.94 | 1.57 | 1.63 | 110.22 |
| FP | 7.92 | 8.59 | 6.38 | 5.64 | 51.56 | 48.98 | 12.54 | 11.40 | 10.87 | 6.79 | 1.29 | 1.47 | 194.75 |
| TN | 3.59 | 3.03 | 11.54 | 8.25 | 140.71 | 140.91 | 24.37 | 23.18 | 14.80 | 11.66 | 3.24 | 3.25 | 504.71 |
| FN | 2.77 | 2.20 | 4.84 | 3.36 | 17.84 | 12.05 | 10.14 | 8.79 | 6.44 | 1.69 | 0.53 | 0.52 | 45.54 |
| MCC | -0.08 | -0.08 | 0.32 | 0.35 | 0.45 | 0.53 | 0.24 | 0.31 | 0.16 | 0.33 | 0.43 | 0.42 | 0.35 |
| FPR | 0.69 | 0.74 | 0.36 | 0.41 | 0.27 | 0.26 | 0.34 | 0.33 | 0.42 | 0.37 | 0.28 | 0.31 | 0.28 |
| FNR | 0.39 | 0.34 | 0.32 | 0.25 | 0.24 | 0.16 | 0.42 | 0.36 | 0.41 | 0.25 | 0.25 | 0.24 | 0.29 |
| (FP/FN) | - | 7.21 | 2.86 | 3.90 | 1.32 | 1.68 | 2.89 | 4.06 | 1.24 | 1.30 | 1.69 | 4.02 | 2.43 |

*Table S2: AutoConfidence outputs for the IAS trained without synthetic errors relative to the gold standard (baseline).*

|  | Lens  L | Lens  R | Lacrimal Gland  L | Lacrimal Gland  R | Orbit  L | Orbit  R | Optic Nerve  L | Optic Nerve  R | Optic  Chiasm | Pituitary  Gland | Cochlea  L | Cochlea  R | Brainstem |
| --- | --- | --- | --- | --- | --- | --- | --- | --- | --- | --- | --- | --- | --- |
| TP | 3.75 | 4.02 | 3.39 | 3.18 | 46.98 | 53.72 | 20.28 | 19.64 | 13.27 | 7.24 | 2.82 | 2.66 | 152.65 |
| FP | 10.21 | 9.16 | 2.8 | 1.79 | 49.13 | 41.95 | 9.7 | 12.37 | 12.58 | 5.4 | 1.3 | 1.48 | 98.66 |
| TN | 3.36 | 3.15 | 3.46 | 4.34 | 156.13 | 157.01 | 23.86 | 21.76 | 12.4 | 11.21 | 1.9 | 2.32 | 568.03 |
| FN | 1.27 | 1.77 | 1.5 | 1.96 | 13.7 | 12.5 | 7.42 | 5.4 | 3.07 | 1.22 | 0.62 | 0.41 | 35.89 |
| MCC | -0.01 | -0.05 | 0.25 | 0.33 | 0.47 | 0.54 | 0.44 | 0.42 | 0.31 | 0.5 | 0.43 | 0.48 | 0.6 |
| FPR | 0.75 | 0.74 | 0.45 | 0.29 | 0.24 | 0.21 | 0.29 | 0.36 | 0.50 | 0.33 | 0.41 | 0.39 | 0.15 |
| FNR | 0.25 | 0.31 | 0.31 | 0.38 | 0.23 | 0.19 | 0.27 | 0.22 | 0.19 | 0.14 | 0.18 | 0.13 | 0.19 |
| (FP/FN) | 8.04 | 5.18 | 1.87 | 0.91 | 3.59 | 3.36 | 1.31 | 2.29 | 4.10 | 4.43 | 2.10 | 3.61 | 2.75 |

*Table S3: AutoConfidence outputs for the IAS trained with synthetic errors relative to the gold standard (baseline).*

|  | Lens  L | Lens  R | Lacrimal Gland  L | Lacrimal Gland  R | Orbit  L | Orbit  R | Optic Nerve  L | Optic Nerve  R | Optic  Chiasm | Pituitary  Gland | Cochlea  L | Cochlea  R | Brainstem |
| --- | --- | --- | --- | --- | --- | --- | --- | --- | --- | --- | --- | --- | --- |
| TP | 4.2 | 5.77 | 4.4 | 3.98 | 50.68 | 62.96 | 26.62 | 26.93 | 23.15 | 9.94 | 3.73 | 3.49 | 201.97 |
| FP | 7.23 | 5.69 | 0.76 | 0.08 | 18 | 10.21 | 1.16 | 1.34 | 0.5 | 0.89 | 0.03 | 0.1 | 22.19 |
| TN | 6.32 | 6.01 | 5.04 | 6.45 | 191.13 | 188.49 | 30.24 | 28.52 | 16.92 | 14.07 | 2.83 | 3.23 | 619.51 |
| FN | 0.83 | 0.64 | 0.95 | 0.75 | 6.13 | 3.52 | 3.24 | 2.37 | 0.75 | 0.18 | 0.06 | 0.05 | 11.57 |
| MCC | 0.28 | 0.41 | 0.69 | 0.85 | 0.75 | 0.87 | 0.86 | 0.88 | 0.94 | 0.91 | 0.97 | 0.96 | 0.9 |
| FPR | 0.53 | 0.49 | 0.13 | 0.01 | 0.09 | 0.05 | 0.04 | 0.04 | 0.03 | 0.06 | 0.01 | 0.03 | 0.03 |
| FNR | 0.17 | 0.10 | 0.18 | 0.16 | 0.11 | 0.05 | 0.11 | 0.08 | 0.03 | 0.02 | 0.02 | 0.01 | 0.05 |
| (FP/FN) | 8.71 | 8.89 | 0.80 | N/A | N/A | N/A | N/A | N/A | N/A | N/A | N/A | N/A | N/A |

*Table S4: AutoConfidence outputs for the IAS relative to the gold standard utilizing the IER and GDC.*

|  | Lens  L | Lens  R | Lacrimal Gland  L | Lacrimal Gland  R | Orbit  L | Orbit  R | Optic Nerve  L | Optic Nerve  R | Optic  Chiasm | Pituitary  Gland | Cochlea  L | Cochlea  R | Brainstem |
| --- | --- | --- | --- | --- | --- | --- | --- | --- | --- | --- | --- | --- | --- |
| TP | 3.38 | 3.63 | 5.81 | 4.06 | 34.88 | 40.39 | 23.93 | 25.53 | 22.36 | 14.38 | 3.95 | 1.82 | 95.45 |
| FP | 2.65 | 1.33 | 0.1 | 0.04 | 23.16 | 18.89 | 2.93 | 2.62 | 1.64 | 0.41 | 0.15 | 0.55 | 73.38 |
| TN | 8.59 | 8.52 | 4.48 | 6.35 | 198.59 | 197.06 | 30.24 | 26.42 | 14.66 | 9.36 | 2.36 | 4.33 | 668.32 |
| FN | 3.95 | 4.62 | 0.76 | 0.82 | 9.31 | 8.85 | 4.16 | 4.59 | 2.67 | 0.93 | 0.18 | 0.16 | 18.08 |
| MCC | 0.24 | 0.34 | 0.85 | 0.85 | 0.62 | 0.68 | 0.77 | 0.76 | 0.79 | 0.89 | 0.89 | 0.77 | 0.63 |
| FPR | 0.24 | 0.14 | 0.02 | 0.01 | 0.10 | 0.09 | 0.09 | 0.09 | 0.10 | 0.04 | 0.06 | 0.11 | 0.10 |
| FNR | 0.54 | 0.56 | 0.12 | 0.17 | 0.21 | 0.18 | 0.15 | 0.15 | 0.11 | 0.06 | 0.04 | 0.08 | 0.16 |
| (FP/FN) | 0.67 | 0.29 | N/A | N/A | 2.49 | 2.13 | N/A | N/A | N/A | N/A | N/A | N/A | 4.06 |

*Table S5: AutoConfidence outputs for the EM-LQ AS relative to the gold standard utilizing the IER and GDC.*

|  | Lens  L | Lens  R | Lacrimal Gland  L | Lacrimal Gland  R | Orbit  L | Orbit  R | Optic Nerve  L | Optic Nerve  R | Optic  Chiasm | Pituitary  Gland | Cochlea  L | Cochlea  R | Brainstem |
| --- | --- | --- | --- | --- | --- | --- | --- | --- | --- | --- | --- | --- | --- |
| TP | 1.33 | 2.73 | 5.3 | 3.6 | 19.6 | 27.34 | 10.18 | 10.53 | 13.06 | 5.07 | 1.46 | 1.32 | 65.54 |
| FP | 5.49 | 4.67 | 0.03 | 0.23 | 37.22 | 34.95 | 9.78 | 7.72 | 5.53 | 3.09 | 1.86 | 0.69 | 83.43 |
| TN | 9.91 | 9.1 | 4.83 | 6.15 | 203.32 | 199.17 | 37.64 | 37.7 | 20.63 | 16.29 | 3.19 | 4.63 | 686.77 |
| FN | 1.85 | 1.6 | 0.98 | 1.28 | 5.8 | 3.72 | 3.65 | 3.21 | 2.1 | 0.63 | 0.12 | 0.22 | 19.5 |
| MCC | 0.05 | 0.25 | 0.83 | 0.73 | 0.44 | 0.55 | 0.47 | 0.55 | 0.63 | 0.65 | 0.47 | 0.67 | 0.52 |
| FPR | 0.36 | 0.34 | 0.01 | 0.04 | 0.15 | 0.15 | 0.21 | 0.17 | 0.21 | 0.16 | 0.37 | 0.13 | 0.11 |
| FNR | 0.58 | 0.37 | 0.16 | 0.26 | 0.23 | 0.12 | 0.26 | 0.23 | 0.14 | 0.11 | 0.08 | 0.14 | 0.23 |
| (FP/FN) | 2.97 | 2.92 | N/A | N/A | 6.42 | 9.40 | 2.68 | 2.40 | 2.63 | 4.90 | 15.50 | 3.14 | 4.28 |

*Table S6: AutoConfidence outputs for the EM-HQ AS relative to the gold standard utilizing the IER and GDC.*

|  | Lens  L | Lens  R | Lacrimal Gland  L | Lacrimal Gland  R | Orbit  L | Orbit  R | Optic Nerve  L | Optic Nerve  R | Optic  Chiasm | Pituitary  Gland | Cochlea  L | Cochlea  R | Brainstem |
| --- | --- | --- | --- | --- | --- | --- | --- | --- | --- | --- | --- | --- | --- |
| TP | 1.51 | 2.57 | 2.69 | 2.66 | 32.05 | 42.8 | 20.16 | 18.06 | 13.02 | 6.73 | 2.96 | 2.67 | 146.27 |
| FP | 9.92 | 8.89 | 2.46 | 1.39 | 36.62 | 30.38 | 7.62 | 10.21 | 10.63 | 4.1 | 0.8 | 0.91 | 77.89 |
| TN | 5.87 | 4.98 | 4.08 | 4.83 | 178.34 | 176.13 | 25.84 | 25.4 | 14.51 | 12.98 | 2.23 | 2.87 | 593.82 |
| FN | 1.28 | 1.66 | 1.92 | 2.38 | 18.92 | 15.88 | 7.64 | 5.48 | 3.16 | 1.27 | 0.66 | 0.41 | 37.26 |
| MCC | -0.06 | -0.03 | 0.2 | 0.32 | 0.41 | 0.54 | 0.5 | 0.47 | 0.38 | 0.57 | 0.56 | 0.62 | 0.64 |
| FPR | 0.63 | 0.64 | 0.38 | 0.22 | 0.17 | 0.15 | 0.23 | 0.29 | 0.42 | 0.24 | 0.26 | 0.24 | 0.12 |
| FNR | 0.46 | 0.39 | 0.42 | 0.47 | 0.37 | 0.27 | 0.27 | 0.23 | 0.20 | 0.16 | 0.18 | 0.13 | 0.20 |
| (FP/FN) | 7.75 | 5.36 | 1.28 | 0.58 | 1.94 | 1.91 | 1.00 | 1.86 | 3.36 | 3.23 | 1.21 | 2.22 | 2.09 |

*Table S7: AutoConfidence outputs for the IAS relative to the gold standard utilizing the IER alone.*

|  | Lens  L | Lens  R | Lacrimal Gland  L | Lacrimal Gland  R | Orbit  L | Orbit  R | Optic Nerve  L | Optic Nerve  R | Optic  Chiasm | Pituitary  Gland | Cochlea  L | Cochlea  R | Brainstem |
| --- | --- | --- | --- | --- | --- | --- | --- | --- | --- | --- | --- | --- | --- |
| TP | 1.6 | 2.56 | 3.05 | 2.26 | 19.45 | 25.02 | 11.2 | 12.11 | 8.93 | 7.13 | 0.97 | 0.51 | 45.44 |
| FP | 4.44 | 2.4 | 2.85 | 1.84 | 38.59 | 34.25 | 15.66 | 16.04 | 15.07 | 7.66 | 3.13 | 1.85 | 123.39 |
| TN | 7.21 | 5.47 | 3.76 | 5 | 192.27 | 185.76 | 29.14 | 24.86 | 13.75 | 8.96 | 2.31 | 4.24 | 660.84 |
| FN | 5.34 | 7.67 | 1.49 | 2.16 | 15.63 | 20.15 | 5.25 | 6.15 | 3.58 | 1.33 | 0.23 | 0.25 | 25.57 |
| MCC | -0.16 | -0.06 | 0.24 | 0.25 | 0.32 | 0.36 | 0.3 | 0.25 | 0.18 | 0.37 | 0.18 | 0.24 | 0.33 |
| FPR | 0.38 | 0.30 | 0.43 | 0.27 | 0.17 | 0.16 | 0.35 | 0.39 | 0.52 | 0.46 | 0.58 | 0.30 | 0.16 |
| FNR | 0.77 | 0.75 | 0.33 | 0.49 | 0.45 | 0.45 | 0.32 | 0.34 | 0.29 | 0.16 | 0.19 | 0.33 | 0.36 |
| (FP/FN) | 0.83 | 0.31 | 1.91 | 0.85 | 2.47 | 1.70 | 2.98 | 2.61 | 4.21 | 5.76 | 13.61 | 7.40 | 4.83 |

*Table S8: AutoConfidence outputs for the EM-LQ AS relative to the gold standard utilizing the IER alone.*

|  | Lens  L | Lens  R | Lacrimal Gland  L | Lacrimal Gland  R | Orbit  L | Orbit  R | Optic Nerve  L | Optic Nerve  R | Optic  Chiasm | Pituitary  Gland | Cochlea  L | Cochlea  R | Brainstem |
| --- | --- | --- | --- | --- | --- | --- | --- | --- | --- | --- | --- | --- | --- |
| TP | 0.62 | 1.4 | 2.64 | 1.77 | 9.53 | 12.34 | 4.34 | 4.41 | 5.49 | 2.12 | 0.34 | 0.4 | 37.23 |
| FP | 6.2 | 6.01 | 2.69 | 2.06 | 47.29 | 49.96 | 15.62 | 13.84 | 13.09 | 6.03 | 2.99 | 1.61 | 111.74 |
| TN | 9.37 | 7.79 | 4.22 | 5.13 | 199.15 | 195.45 | 36.66 | 37.17 | 19.7 | 15.99 | 3.17 | 4.55 | 677.48 |
| FN | 2.39 | 2.91 | 1.59 | 2.3 | 9.97 | 7.44 | 4.63 | 3.74 | 3.03 | 0.94 | 0.15 | 0.3 | 28.78 |
| MCC | -0.15 | -0.1 | 0.23 | 0.15 | 0.19 | 0.26 | 0.14 | 0.2 | 0.2 | 0.29 | 0.11 | 0.21 | 0.3 |
| FPR | 0.40 | 0.44 | 0.39 | 0.29 | 0.19 | 0.20 | 0.30 | 0.27 | 0.40 | 0.27 | 0.49 | 0.26 | 0.14 |
| FNR | 0.79 | 0.68 | 0.38 | 0.57 | 0.51 | 0.38 | 0.52 | 0.46 | 0.36 | 0.31 | 0.31 | 0.43 | 0.44 |
| (FP/FN) | 2.59 | 2.07 | 1.69 | 0.90 | 4.74 | 6.72 | 3.37 | 3.70 | 4.32 | 6.41 | 19.93 | 5.37 | 3.88 |

*Table S9: AutoConfidence outputs for the EM-HQ AS relative to the gold standard utilizing the IER alone.*

|  | Lens  L | Lens  R | Lacrimal Gland  L | Lacrimal Gland  R | Orbit  L | Orbit  R | Optic Nerve  L | Optic Nerve  R | Optic  Chiasm | Pituitary  Gland | Cochlea  L | Cochlea  R | Brainstem |
| --- | --- | --- | --- | --- | --- | --- | --- | --- | --- | --- | --- | --- | --- |
| TP | 12.52 | 11.81 | 6.14 | 4.95 | 93.22 | 95 | 29.95 | 31.88 | 25.84 | 12.54 | 4.12 | 4.13 | 249 |
| FP | 1.44 | 1.37 | 0.05 | 0.01 | 2.89 | 0.67 | 0.03 | 0.13 | 0.02 | 0.11 | 0 | 0 | 2.32 |
| TN | 4.08 | 4.26 | 4.66 | 5.86 | 168.07 | 168.22 | 28.49 | 25.01 | 14.79 | 12.3 | 2.47 | 2.68 | 593.24 |
| FN | 0.54 | 0.66 | 0.3 | 0.44 | 1.75 | 1.29 | 2.79 | 2.15 | 0.67 | 0.14 | 0.06 | 0.05 | 10.69 |
| MCC | 0.74 | 0.73 | 0.94 | 0.92 | 0.96 | 0.98 | 0.91 | 0.92 | 0.96 | 0.98 | 0.98 | 0.98 | 0.96 |
| FPR | 0.26 | 0.24 | 0.01 | 0.00 | 0.02 | 0.00 | 0.00 | 0.01 | 0.00 | 0.01 | 0.00 | 0.00 | 0.00 |
| FNR | 0.04 | 0.05 | 0.05 | 0.08 | 0.02 | 0.01 | 0.09 | 0.06 | 0.03 | 0.01 | 0.01 | 0.01 | 0.04 |
| (FP/FN) | N/A | N/A | N/A | N/A | N/A | N/A | N/A | N/A | N/A | N/A | N/A | N/A | N/A |

*Table S10: AutoConfidence outputs for the IAS relative to the gold standard utilizing the GDC alone.*

|  | Lens  L | Lens  R | Lacrimal Gland  L | Lacrimal Gland  R | Orbit  L | Orbit  R | Optic Nerve  L | Optic Nerve  R | Optic  Chiasm | Pituitary  Gland | Cochlea  L | Cochlea  R | Brainstem |
| --- | --- | --- | --- | --- | --- | --- | --- | --- | --- | --- | --- | --- | --- |
| TP | 6.56 | 6.29 | 6.26 | 4.64 | 78.97 | 78.87 | 32.56 | 32.95 | 26.21 | 15.83 | 4.16 | 2.92 | 222.11 |
| FP | 1.4 | 0.74 | 0.03 | 0.01 | 8.06 | 5.29 | 0.24 | 0.48 | 0.23 | 0.15 | 0.06 | 0.15 | 25.33 |
| TN | 7.81 | 8.32 | 4.25 | 5.93 | 172.83 | 176.22 | 24.07 | 21.08 | 12.32 | 8.3 | 2.23 | 3.6 | 593.16 |
| FN | 2.82 | 2.74 | 0.61 | 0.68 | 6.08 | 4.8 | 4.4 | 4.65 | 2.56 | 0.81 | 0.18 | 0.19 | 14.64 |
| MCC | 0.55 | 0.63 | 0.89 | 0.88 | 0.88 | 0.91 | 0.85 | 0.83 | 0.86 | 0.92 | 0.92 | 0.9 | 0.89 |
| FPR | 0.15 | 0.08 | 0.01 | 0.00 | 0.04 | 0.03 | 0.01 | 0.02 | 0.02 | 0.02 | 0.03 | 0.04 | 0.04 |
| FNR | 0.30 | 0.30 | 0.09 | 0.13 | 0.07 | 0.06 | 0.12 | 0.12 | 0.09 | 0.05 | 0.04 | 0.06 | 0.06 |
| (FP/FN) | 0.50 | 0.27 | N/A | N/A | N/A | N/A | N/A | N/A | N/A | N/A | N/A | N/A | N/A |

*Table S11: AutoConfidence outputs for the EM-LQ AS relative to the gold standard utilizing the GDC alone.*

|  | Lens  L | Lens  R | Lacrimal Gland  L | Lacrimal Gland  R | Orbit  L | Orbit  R | Optic Nerve  L | Optic Nerve  R | Optic  Chiasm | Pituitary  Gland | Cochlea  L | Cochlea  R | Brainstem |
| --- | --- | --- | --- | --- | --- | --- | --- | --- | --- | --- | --- | --- | --- |
| TP | 8.55 | 8.55 | 6.08 | 4.62 | 76.45 | 79.14 | 27.32 | 25.64 | 21.91 | 10.37 | 3.16 | 2.81 | 196.69 |
| FP | 2.1 | 1.86 | 0.04 | 0.01 | 12.93 | 13.02 | 2.67 | 2.15 | 0.72 | 1.01 | 0.87 | 0.11 | 32.65 |
| TN | 6.78 | 6.7 | 4.33 | 5.84 | 172.2 | 170.11 | 27.43 | 27.98 | 16.75 | 13.21 | 2.46 | 3.71 | 609.03 |
| FN | 1.16 | 1 | 0.7 | 0.79 | 4.35 | 2.91 | 3.84 | 3.39 | 1.94 | 0.48 | 0.15 | 0.23 | 16.87 |
| MCC | 0.65 | 0.68 | 0.87 | 0.87 | 0.85 | 0.87 | 0.79 | 0.81 | 0.87 | 0.88 | 0.71 | 0.9 | 0.85 |
| FPR | 0.24 | 0.22 | 0.01 | 0.00 | 0.07 | 0.07 | 0.09 | 0.07 | 0.04 | 0.07 | 0.26 | 0.03 | 0.05 |
| FNR | 0.12 | 0.10 | 0.10 | 0.15 | 0.05 | 0.04 | 0.12 | 0.12 | 0.08 | 0.04 | 0.05 | 0.08 | 0.08 |
| (FP/FN) | 1.81 | 1.86 | N/A | N/A | N/A | N/A | N/A | N/A | N/A | N/A | N/A | N/A | N/A |

*Table S12: AutoConfidence outputs for the EM-HQ AS relative to the gold standard utilizing the GDC alone.*

- **Figures:**


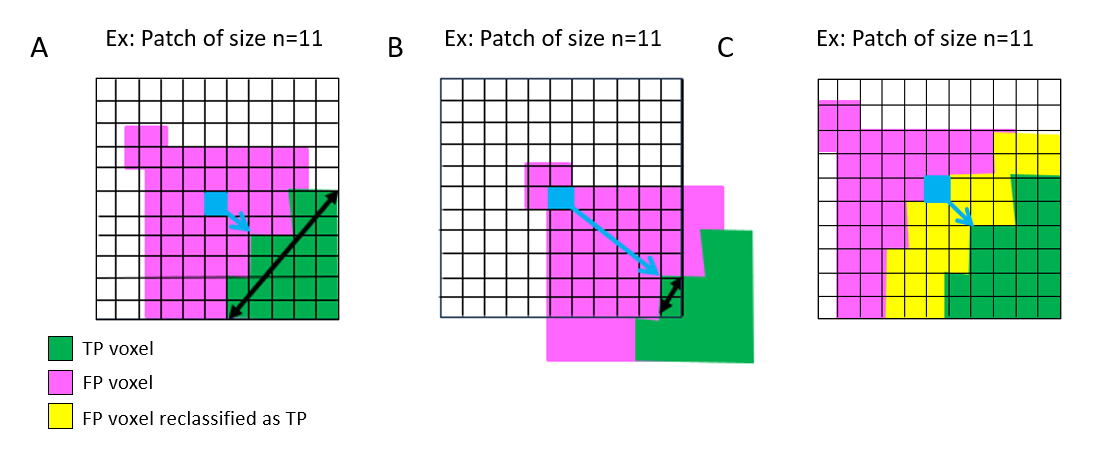


*Figure S1: a and b) Illustration of GDC in different scenarios. The voxel under consideration is the central blue voxel. FP voxels are represented in pink, TP voxels in green. A) Example conversion of a FP voxel to TP. Blue arrow (D(fp_tp)) is shorter than the black arrow (D(tp)_max). B) A voxel which does not qualify for conversion, as it lies too far from the TP region (blue arrow longer than black arrow). C) The final classification, showing converted voxels in yellow.*

*
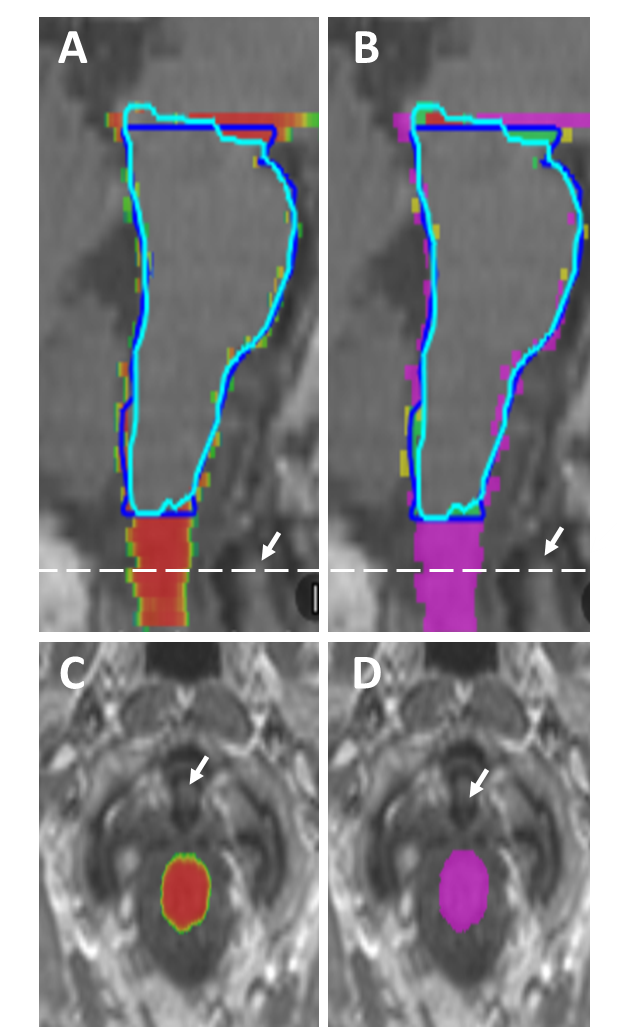
*

*Figure S2: a) Sagittal (dotted line represents axial slice), and c) axial T1w-Gd MRI showing ACo overprediction of uncertainty beyond the inferior limit of brainstem, resulting in false-positives. Blue represents the gold standard contours; light blue represents EM-HQ AS. The white arrows show the tip of the dens of C2, the anatomical definition of the inferior limit of brainstem. b) Sagittal and d) axial four-colour-map showing regions of TP (green), FP (pink) and GDC modified FP (yellow) relative to the differences to gold-standard, for the ACo prediction.*
